# Microencapsulation of a Pickering Oil/Water Emulsion Loaded with Vitamin D3

**DOI:** 10.3390/gels9030255

**Published:** 2023-03-22

**Authors:** Alessandro Candiani, Giada Diana, Manuel Martoccia, Fabiano Travaglia, Lorella Giovannelli, Jean Daniel Coïsson, Lorena Segale

**Affiliations:** Department of Pharmaceutical Sciences, University of Piemonte Orientale, Largo Donegani 2, 28100 Novara, Italy; alessandro.candiani@uniupo.it (A.C.); giada.diana@uniupo.it (G.D.); manuel.martoccia@uniupo.it (M.M.); fabiano.travaglia@uniupo.it (F.T.); lorella.giovannelli@uniupo.it (L.G.); jeandaniel.coisson@uniupo.it (J.D.C.)

**Keywords:** vitamin D3, sodium alginate, microparticles, ionotropic gelation

## Abstract

The ionotropic gelation technique was chosen to produce vitamin D3-loaded microparticles starting from oil-in-water (O/W) Pickering emulsion stabilized by flaxseed flour: the hydrophobic phase was a solution of vitamin D3 in a blend of vegetable oils (ω6:ω3, 4:1) composed of extra virgin olive oil (90%) and hemp oil (10%); the hydrophilic phase was a sodium alginate aqueous solution. The most adequate emulsion was selected carrying out a preliminary study on five placebo formulations which differed in the qualitative and quantitative polymeric composition (concentration and type of alginate selected). Vitamin D3-loaded microparticles in the dried state had a particle size of about 1 mm, 6% of residual water content and excellent flowability thanks to their rounded shape and smooth surface. The polymeric structure of microparticles demonstrated to preserve the vegetable oil blend from oxidation and the integrity of vitamin D3, confirming this product as an innovative ingredient for pharmaceutical and food/nutraceutical purposes.

## 1. Introduction

Vitamin D is a fat-soluble compound that plays an important role in the human body, as it is involved in several metabolic processes such as calcium and phosphorus metabolism, skeletal and neuromuscular homeostasis [1]. Vitamin D is obtained endogenously by UV exposure or by specific food intake. In nature, it can be found in two forms, D2 (ergocalciferol) and D3 (cholecalciferol). Vitamin D2 derives from ergosterol mushrooms and yeast after UV irradiation, while vitamin D3 (vit D3) is present in foods of animal origin (eggs, fish and meat) and it is also synthesized within human skin thanks to UV sunlight exposure [2].

Although there are natural sources of vitamin D, they are often not enough to satisfy the required amount for human health. Vitamin D deficiency is generally caused by several factors, including lack of sun exposure, lifestyle, poor diet, aging, and some health states. Recently, there is much evidence about vitamin D deficiency, above all for the population of the countries of the northern hemisphere because of their insufficient sunlight exposure. It results that in several European countries, more than 50% of the adult population is affected by vitamin D deficiency. In particular, about 40% of people are vitamin D deficient and 13% are severely deficient [3,4]. Many pharmaceutical supplements (oil solutions, capsules, tablets) are commercially available to overcome this problem. In general, patient compliance with these products may be not fully satisfying because they require daily administration for most of the year. Starting from these assumptions, a good alternative could be represented by the intake of foods enriched with vitamin D. One of the major problems associated with the incorporation of vit D3 directly into foods is its rapid degradation due to light, oxygen and heat sensitivity [5]. Moreover, it is a highly hydrophobic molecule characterized by low water solubility and poor oral bioavailability.

For these reasons, the encapsulation of vitamin D could be a good strategy to obtain a food ingredient able to favor vitamin administration and improve its efficacy and stability. In the literature, many examples are reported [5] and the inclusion of vitamin D in emulsions or microparticulate systems is the most frequent proposal [6,7,8].

Microencapsulation is a useful process to entrap one or more solid, liquid or gaseous active substances into small solid microparticles (microcapsules or microspheres) to preserve these substances and to release them as needed [9,10]. The obtainment of a product of a high-quality level depends on the selection of the formulation components and the most adequate production technique. For vitamin D, it is mandatory to preserve its characteristics during the treatment, so it is important to avoid technologies involving the use of high temperatures or organic and toxic solvents. Coacervation or ionotropic gelation could be a good choice [11,12]. For ionotropic gelation, sodium alginate, a polymer extracted from marine brown algae, is selected as wall material thanks to its ability to gel when in contact with a divalent cation solution. It is possible to dissolve or disperse an active ingredient in the polymeric solution and to dispense it drop by drop in the gelation bath, obtaining loaded particles [13]. The microencapsulation of hydrophilic compounds is quite difficult because it is often associated with some drawbacks and disadvantages, such as the diffusion of the active compound out of the polymeric network during the production step, a not-so-high encapsulation efficiency and a fast release of the encapsulated compound. On the other hand, when the goal is the microencapsulation of a hydrophobic drug, it is necessary to select the most advantageous approach to preserve the system stability. A valid solution could be the production of core–shell systems, in which the hydrophobic drug is loaded into the core and the alginate is the polymer that composes the shell. Another possibility could be the combination of emulsification and gelation process, that is the dissolution of the drug in the hydrophobic phase of an oil in water (O/W) emulsion and the entrapment of this emulsion in alginate beads [14,15,16]. In this work, the active compound is vitamin D, and the final product is intended to be a food ingredient, so it is important to pay attention to the oil selected as a solvent for the vitamin and to the type of emulsifier used to stabilize the emulsion before its microencapsulation treatment.

In the last years, Pickering emulsions, which are composed of two immiscible liquids stabilized at the interface by solid particles, have found a growing interest linked to the important advantages that they offer. Pickering emulsions are more stable, biocompatible and less cytotoxic than conventional surfactant-based emulsions [17]. The solid particles used as stabilizers can be inorganic (hydroxyapatite, silica, etc.) even if today the attention focuses on natural solid emulsifiers such as starch, zein, soy protein and whey protein [18].

This research work aimed to obtain vit D3-loaded microparticles starting from a Pickering O/W emulsion to combine the high encapsulation performance of ionotropic gelation with the several advantages of this type of preparation.

In detail, an O/W emulsion, in which the hydrophobic phase was a solution of vit D3 in oil and the hydrophilic phase was a sodium alginate aqueous solution, has been added dropwise in CaCl_2_ aqueous solution to obtain microparticulate systems suitable as an ingredient for the production of food enriched with vit D3. Flaxseed flour was used as emulsion stabilizer. The main goal of this research was the entrapment of the vitamin D3 oily solution into a microparticulate system in order to facilitate its use and improve its stability. The choice to identify a Pickering emulsion as the best starting point instead of a conventional emulsion was linked to the necessity to have a formulation as much as possible poor in synthetic compounds and useful for food applications.

To improve the added value of the final microencapsulated product, the oil in which vit D3 has been solubilized was a blend of vegetable oil characterized by a well-defined ω6:ω3 ratio (4:1). The rationale of this choice is attributable to the central role of polyunsaturated fatty acids in human health associated to their anti-inflammatory and cardiovascular protective action. Among these fatty acids, ω3 and ω6 are the most important and they must be introduced into the diet because they are not synthesized by the human organism [19,20].

## 2. Results and Discussion

### 2.1. Vegetable Oil Blend Characterization: Evaluation of Fatty Acids Composition, ω6:ω3 Ratio and Peroxide Value

The analysis of the triglyceride acid component was carried out by gas chromatography (GC) coupled with a flame ionization detector (FID) on the methyl esters of fatty acids and allowed to obtain information on the lipid composition of the oil selected for this work (Table 1). The most abundant fatty acids were respectively oleic acid (C18:1), linoleic acid (C18:2 n6cis) and palmitic acid (C16:0) and the ω6:ω3 ratio calculated by the experimental results (4.5:1) confirmed the theoretical one (4:1). The obtained results are a demonstration of the good nutritional intake of the selected vegetable oil blend.

Peroxide values obtained on the vegetable oil blend are reported in Table 2. As expected, the different methods (iodometric and spectrophotometric) gave different results according to their specificity.

### 2.2. Preliminary Study

The starting phase of this work focused on the identification of the best quali-quantitative composition of the emulsion to submit to the microencapsulation process. Five placebo emulsions were prepared and characterized. Three types of sodium alginate with different viscosity were selected: Protanal LF10/60 (low viscosity grade), Manucol (medium viscosity grade) and sodium alginate Farmalabor (high viscosity grade). They were used in the same concentration (OL-1, OL-4 and OL-5 formulation) to compare their performance as carrier excipients in the microparticle formation. Moreover, in the case of the least viscous polymer, its concentration in the emulsion composition has been increased to verify if this variation could be responsible for better results in the microparticle production process.

**Figure 1:**
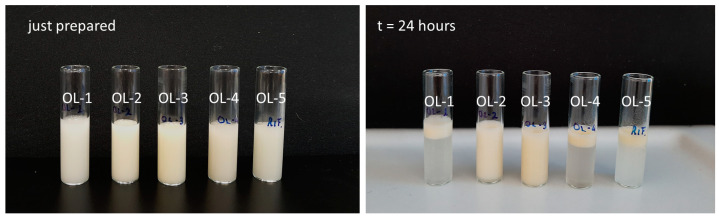
Images of the placebo emulsions immediately after the preparation and after 24 h.

The emulsion represents the starting material for the encapsulation treatment. Therefore, it is mandatory to verify if it has all the characteristics to be submitted to the process. First of all, the emulsion has to be stable for a time period sufficient to conclude the microencapsulation process. Immediately after the preparation, all the emulsions were stable. They appeared milky and opaque even if those in which the polymer concentration was higher (OL-2 and OL-3) presented a yellow color. OL-4 emulsion started to separate just 1 h after the preparation, while the stability of the other samples was maintained over time. After 24 h, only OL-2 and OL-3 formulations could be considered stable because they did not show any phase separation (Figure 1).

Furthermore, the viscosity of the material to be treated was an important aspect to consider, because it could affect its workability and some characteristics of the final product as, for example, its particle size: the lower the viscosity of the starting material, the smaller the diameter of the obtained microparticles [21] will probably be. The results reported in Table 3 confirmed that the viscosity of the different placebo emulsions changed according to the type of polymer used and its concentration in the formulation. OL-1 and OL-4 emulsions had very low viscosity, on the contrary, the too-high viscosity of the OL-3 emulsion probably made this system unsuitable as starting material to be processed.

**Figure 2:**
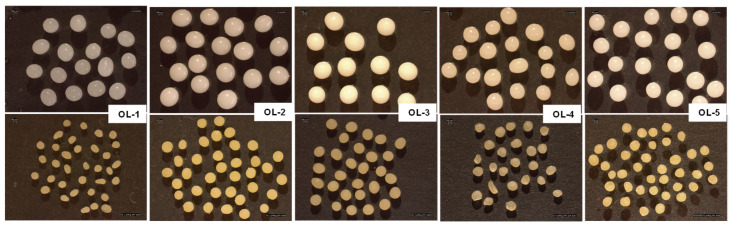
Stereomicroscope images of wet (first line) and dried (second line) placebo microparticles.

The placebo emulsions were used to prepare small batches of microparticles by ionotropic gelation (with a simple dripping tool) to verify significant differences among the batches. All the resulting formulations were suitable for this purpose, different types of microparticles were obtained (Figure 2) and each of them was characterized.

Immediately after the preparation, wet microparticles were quite large in dimension (about 3.0 mm in diameter), white and glossy with a smooth and homogeneous surface (Figure 2). OL-2, OL-3 and OL-5 systems were also regular in shape, while OL-1 and OL-4 batches included many irregular and elongated particles. After drying, in general, the diameters of the microparticles decreased because of the water evaporation and the packing of the polymer chains, and in some cases, the shape irregularity became evident, as confirmed by the stereomicroscope images and by the shape factor values which are very far from that assumed by a spherical particle (Table 3).

OL-1 and OL-4 emulsions were discarded because the obtained microparticles were inhomogeneous in dimensions and irregular in shape, moreover, OL-4 emulsion was also quite unstable. OL-3 formulation was excluded because its viscosity was too high, and this could be a problem when using the Encapsulator instrument to scale up microparticle production. The results of this preliminary study allowed the identification of OL-2 as the most suitable formulation to be processed (stereomicroscope image of this emulsion is reported as Supplementary Material, Figure S1) and it has been preferred to OL-5 because its lower viscosity represented an advantage for its processability.

### 2.3. Placebo and vit D3-Loaded Microparticles

OL-2 emulsion was used to produce placebo and vit D3-loaded microparticles by ionotropic gelation, using the prilling vibration technique.

**Figure 3:**
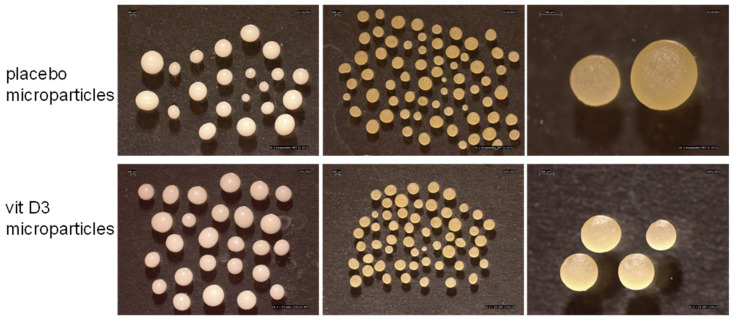
Stereomicroscope images of wet and dried microparticles.

In both cases, immediately after the preparation, microparticles were characterized by a smooth surface and an almost spherical shape (Figure 3). The drying process was responsible for some modifications in the morphological appearance of microparticles: their color changed from white to yellow, their dimensions decreased due to a volume shrinkage that occurred by evaporation of the water, while the smooth and shiny surface was maintained, and the regular shape was retained.

Wet microparticles were milky because they assumed the colour of the loaded emulsion. After drying, during which water evaporated, the content of microparticles was represented principally by oil blend (placebo systems) or vit D3 oily solution and the colour of the systems turned to glossy yellow.

Wet microparticles were quite large in dimension. Placebo systems had a diameter close to 1.8 mm, which slightly increased up to 2 mm in the case of vit D3-loaded systems (Table 4). The shape factor values, very close to 1, confirmed what was observed by the stereomicroscope images, i.e., the obtainment of very regular in shape particles.

In general, upon drying, microparticles had remarkably reduced their size up to about 37% and 45% for placebo and vit D3 systems respectively. The average diameter of dried placebo microparticles was 1121 µm ± 240 and that of vit D3 systems was 1092 µm ± 163. However, the diameter variation did not affect the shape of the particles, so the shape factor values did not change before and after drying (Table 4).

**Figure 4:**
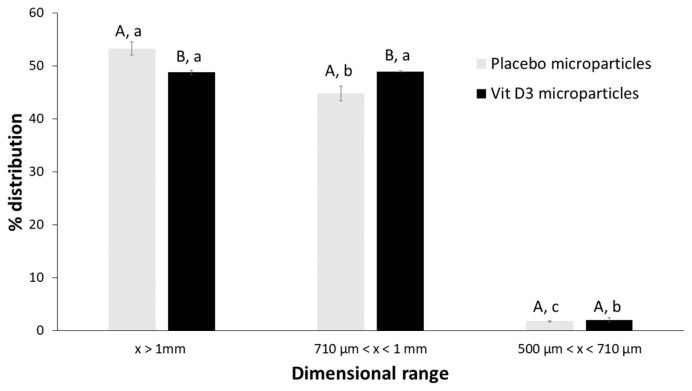
Particle size distribution of dried microparticles. Dissimilar capital letters over bars denote significant differences between samples within the same dimensional range (*p* < 0.05); while different lowercase alphabetical characters denote significant differences among different dimensional ranges within the same sample (*p* < 0.05).

Particle size distribution of dried microparticles has been reported in Figure 4. In both batches of microparticles, most of the units were greater than 710 µm. In particular, in the case of placebo systems, the size of over 54% of particles was more than 1 mm while that of about 44% of particles was between 710 µm and 1 mm. For vit D3-loaded systems, there was an equal distribution of units (about 49%) into the two size ranges (over 1 mm and between 710 µm and 1 mm).

The drying process to which microparticles were submitted was effective: the residual humidity determined was about 6% (Table 5), attributable to the free and bounded water loss.

The rounded shape and the smooth surface of dried microparticles were responsible for a small angle of repose, associated with excellent flowability properties (Table 5). This is important because the particulate products can be transported using only the force of gravity or little energy: this represents an added value and a great advantage for the subsequent processing or handling phases to which they may be subjected.

**Figure 5:**
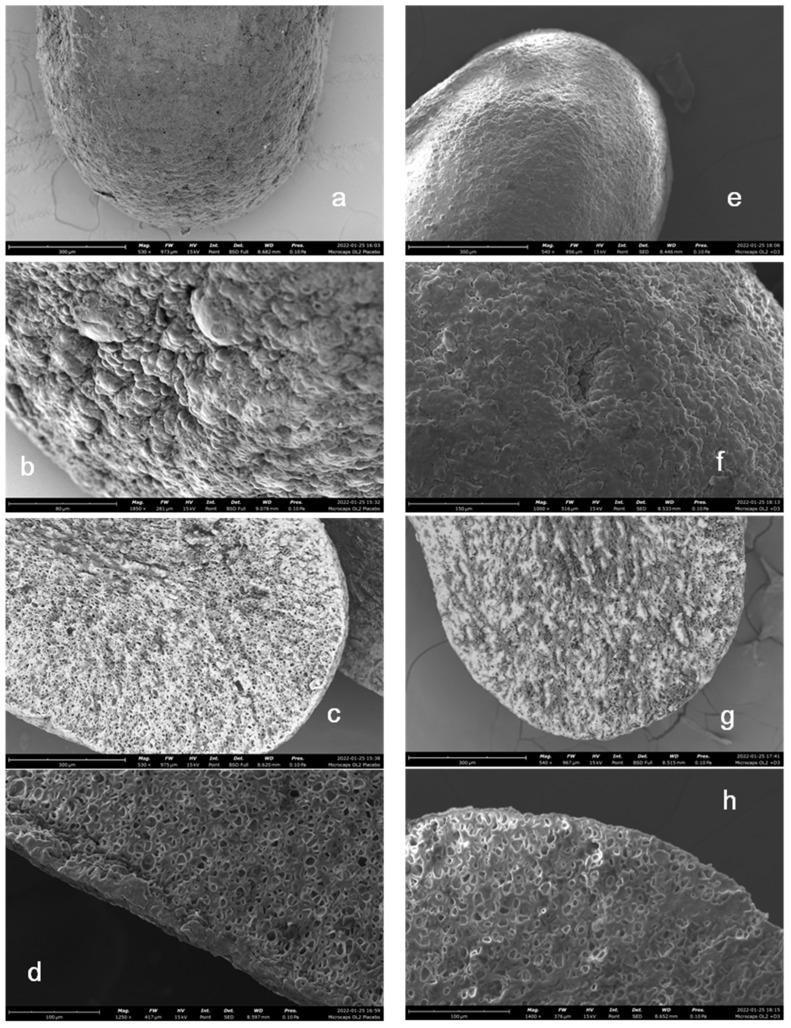
SEM images of the external surface ((**a**,**b**), magnification respectively 530× and 1850×) and a cross-section ((**c**,**d**), magnification respectively 530× and 1250×) of placebo microparticles; external surface ((**e**,**f**), magnification respectively 540× and 1000×) and a cross-section ((**g**,**h**), magnification respectively 540× and 1400×) of vit D3-loaded systems.

The external structure and a cross-section of the dried multi-unit systems were evaluated by SEM. There were no evident differences between placebo and vit D3 microparticles: in both cases, the external surface was homogeneous, continuous, without cracks nor fractures (Figure 5a,b,e,f). In the same way, the internal surface of microparticles was also quite similar. In both cases, it was characterized by a non-compact structure. There were many little porosities, typical of the cross-linked nature of the systems, and the structure was characterized by a high-density polymeric network (Figure 5c,d,g,h).

The oil content of dried microparticles was defined by a liquid–liquid extraction method. It was characterized by an extraction efficiency of 90% when applied directly to the starting emulsion and 84.04% ± 5.15 when used on microparticles. According to the extraction results, the experimental oil content of placebo and vit D3 microparticles was quite high, respectively 56.45% ± 4.43 and 53.44% ± 1.42, even if lower than the theoretical one (about 69%). This result depends on various factors, above all on the efficiency of the extraction method but also on the eventual oil loss during the production of microparticles or their disintegration before the extraction.

The peroxide value of the extracted oil was significantly higher than that of the fresh oil (Table 2). This result was unexpected because in general microencapsulation should give more protection against degradation, oxidation, etc. For this reason, to establish if this result was due to the stress of the microencapsulation process or the extraction method, the peroxide value was determined also on the fresh vegetable oil mixture after submission to a simulated liquid-liquid extraction process. The obtained results demonstrate that the treatment of the vegetable oil blend with organic solvents (hexane and acetone) was responsible for its rapid oxidation (Table 2).

In a matrix-type drug delivery system, the active substance must be homogeneously dispersed in the polymeric carrier to guarantee a good drug release performance because a heterogeneous distribution of the active principle could lead to undesired burst release or degradation of the drug excessively exposed to the external environment. In this study, it was mandatory that the vit D3 oily solution present on the microparticle surface was quite low, primarily to limit vitamin light degradation. The excellent flowability properties of the microparticulate systems were already a good indication that the oil had been well encapsulated, but this result was further strengthened by the percentage of oil recovered from the microparticle surface that did not exceed 3% (2.43% ± 0.52).

Vit D3 recovered from the oily solution extracted from microparticles was 78.37% ± 8.54 of the loaded amount. This result could be ascribed to a loss of vitamin during the production of microparticles, during the liquid–liquid oil extraction, but also to the efficiency of the solid phase extraction (SPE) method. SPE efficiency was defined by submitting an oily vit D3 solution to the same process immediately after preparation. In this case, vit D3 recovery was 73.67% ± 10.57.

In general, when dried alginate microparticles are put in contact with an aqueous fluid, they absorb it, hydrate and swell. The systems may undergo disintegration/dissolution according to the fluid nature and the presence of some ions in the fluid (in particular Na^+^ ions) [22].

**Figure 6:**
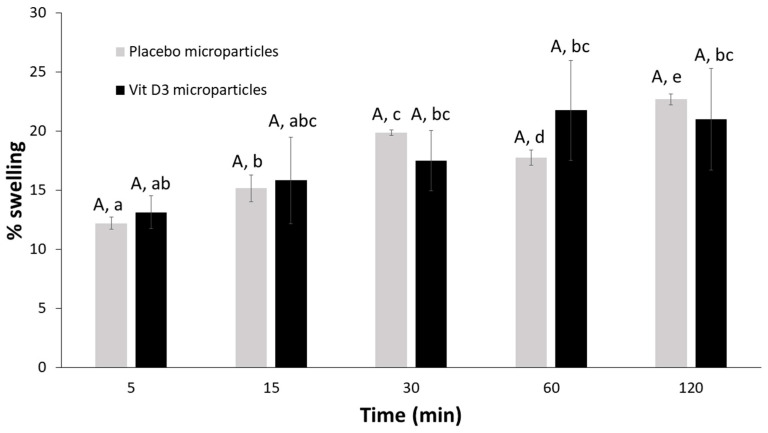
Swelling percentages of dried microparticles in water. Different capital letters over bars show significant differences between samples at the same time intervals (*p* < 0.05); while dissimilar lowercase letters indicate significant differences among different time intervals within the same sample (*p* < 0.05).

The swelling behavior of dried placebo and vit D3-loaded microparticles was very similar: after 5 min in water, microparticles started to swell even if the increase of their weight was quite limited and the swelling ratio did not exceed 15%. The swelling process went ahead without microparticles disintegration, although the water uptake increased slowly during all the tests reaching only 21–22% as the maximum swelling point at the end of the test (Figure 6). This result may be in contrast with others reported in the literature because generally dried alginate microparticles can absorb much larger quantities of water [12]. The reason for this very limited swelling behavior could be attributable to the reduced affinity between microparticulate systems and water. Even if the polymeric carrier responsible for the tridimensional structure of both placebo and vit D3-loaded microparticles was hydrophilic, an important quantity of lipophilic compounds was included in the formulations, that is, vegetable oil blend alone or the vit D3 solution in the vegetable oil blend. The oily phase was homogeneously dispersed in the polymeric matrix, around the alginate chains and in the porosity of the polymeric network, giving a purely lipophilic character to the system, which was probably the reason why it was not able to absorb considerable amounts of water.

To verify if during the swelling process microparticles lost a portion of their oily content, at the end of the test they were dried in an oven at 40 °C for 2 h and after that re-weighed. During this drying, process water evaporated again, and the systems completely recovered their initial weight demonstrating that no oily phase was released during their swelling in water.

## 3. Conclusions

Ionotropic gelation turned out to be a good technique for vit D3 microencapsulation. The combination of the advantages of Pickering emulsions and ionotropic gelation technique allowed to obtain vit D3-loaded microparticles with a resistant structure, good appearance and great biocompatibility.

The obtained microparticles in the dried state were about 1 mm in size, regular in shape and characterized by good flowability. The polymeric structure of microparticles could protect the vegetable oil mixture from oxidation and preserve quite well the integrity of vit D3.

In particular, the data collected confirm the good quality of the obtained product and the possibility of using it as an innovative ingredient for pharmaceutical and food/nutraceutical purposes.

## 4. Materials and Methods

### 4.1. Materials

A blend of vegetable oils (ω6:ω3, 4:1) composed of extra virgin olive oil (90%) and hemp oil (10%) has been produced by F.lli Ruata S.p.A (Baldissero D’Alba, Cuneo, Italy). Different types of sodium alginate have been used: sodium alginate (mannuronic (M) and guluronic (G) residues ratio 1.8–2.2, viscosity 1% solution 500–600 mPa·s, Farmalabor, Assago, Milan, Italy); Protanal LF 10/60 (M/G ratio 1.50, viscosity 1% solution 20–70 mPa·s, Dupont Italia, Milan, Italy) and Manucol LKX 50 DR (M/G ratio 1.44, viscosity 1% solution 60–170 mPa·s, Dupont, Milan, Italy).

Vit D3 has been purchased by Alfa Aesar, (Thermo Fisher GmbH, Kandel, Germany); flaxseed flour (HI-SMOOTH) has been supplied by HIFOOD (Parma, Italy); calcium chloride was purchased from Sigma-Aldrich (Milan, Italy).

All other reagents were of analytical grade and used as received.

### 4.2. Experimental Methods

#### 4.2.1. Vegetable Oil Blend Characterization: Evaluation of ω6:ω3 Ratio

Fatty acid methyl esters (FAMEs) were obtained by transesterification of triglycerides (200 μL) as described by Locatelli et al. (2011) [23].

FAMEs were analyzed on a Thermo Trace 1300 Gas Chromatograph (GC) equipped with a flame ionization detector (FID) and a split-splitless injector, using a DB23 column (30 m, inner diameter of 0.25 mm, and film thickness of 0.25 μm; J & W Scientific). Hydrogen was used as the gas carrier, with a flux of 1.5 mL/min. The injector and the detector were operated at 250 °C and 350 °C, respectively, and the temperature ramp was 5 °C/min. The identification was obtained by comparing the retention times obtained from a mixture of 37 FAME standards (Supelco).

#### 4.2.2. Determination of Peroxide Value of Vegetable Oil Blend

These tests were carried out on both a fresh vegetable oil blend and the oil extracted from dried microparticles. In particular, peroxidation parameters were determined by two different methods (iodometric and spectrophotometric methods) on the fresh oil and only by the spectrophotometric method on the oil extracted from microparticles because of the reduced amount of available sample.

The spectrophotometric method is based on the ability of hydroperoxides to oxidize ferrous ions (Fe^2+^) to ferric ions (Fe^3+^) in an acidic medium [24]. In detail, an exacted weighed amount of oily sample (included between 0.01 and 0.30 g) was introduced in a vial and 9.9 mL chloroform-methanol (7:3, *v*/*v*) mixture and 50 μL ammonium thiocyanate solution (30% *w*/*w*) were added and mixed. The sample was vortexed for 5 s, then, 50 μL of iron(II) chloride solution (2 mg/mL acidified with 10 M HCl) was added. The sample was vortexed for 5 s and incubated for 5 min at room temperature in the dark and then the absorbance of the sample was determined spectrophotometrically at 500 nm (Shimadzu UV-1900). The iodometric method is based on the reaction between a saturated solution of potassium iodide and an oil sample following the ability of hydroperoxides to oxidize iodide ions (I^−^) to iodine (I_2_). 3 g of oil was diluted in a 25 mL solvent mixture (acetic acid and dichloromethane 3:2, *v*/*v*) and 0.5 mL of saturated KI solution was added. The sample was gently shaken for 1 min, stored in the dark for 5 min, and diluted with 75 mL of distilled water. Titration was carried out against 0.01 N Na_2_S_2_O_3_ using as indicator a 1% starch solution [25].

#### 4.2.3. Preliminary Studies

The preliminary study was carried out for the identification of the best formulation to be submitted to the microencapsulation process. This step of the work was focused only on placebo formulations, that is formulations without vit D3.

In detail, five placebo oil in water (O/W) emulsions have been prepared. They differed in the qualitative and quantitative composition, in particular in the concentration and type of alginate selected (Table 6), causing an impact on the morphology and particle size of the final product [21].

For the preparation of each emulsion, sodium alginate was dissolved in water under magnetic stirring, then flaxseed flour, the stabilizer of the final emulsion, was solubilized into the polymeric solution. The vegetable oil blend was added, and the two phases were emulsified by Ultra-Turrax for a few minutes.

Each emulsion was characterized regarding viscosity, stability and processability.

The viscosity was measured at room temperature using a Brookfield viscometer (Brookfield Programmable DV-II Viscometer) equipped with an S18 spindle. The stability of the emulsions was evaluated in the first 24 h after the preparation: an aliquot of each emulsion has been transferred into a glass tube, maintained at room temperature, visually inspected at predefined time intervals (1 h, 2 h) and photographed after 24 h to establish if the two phases were separated or not. The last step of the preliminary study was carried out to verify if all five formulations were adequate to be submitted to ionotropic gelation. The emulsions were manually dripped, through a needle (800 µm in diameter), into a CaCl_2_ solution (100 mM) where the drops were immediately transformed into gel microparticles. After curing, microparticles were recovered by filtration, washed with deionized water to eliminate the calcium excess on their surface and dried in an oven at 40 °C overnight. All the samples were characterized in morphology and dimensions to underline possible differences among them.

#### 4.2.4. Preparation of Placebo and vit D3-Loaded Microparticles

The formulation of the emulsion identified as the most satisfying in the preliminary studies was the starting material for the production of placebo microparticles (containing only the blend of vegetable oils without vit D3) and vit D3-loaded microparticles (Table 7).

Microparticles were prepared by ionotropic gelation using the prilling vibration technique (Encapsulator B-390, Buchi, Flawil, CH): a laminar-flow fluid jet was subjected to a superimposed mechanical vibration, responsible for its division into regular-sized droplets. The emulsion was prepared as described in the previous section: for placebo formulation, the oily phase included only the vegetable oil blend, while in the case of vit D3-loaded systems, the oily phase was a solution of vit D3 in the vegetable oil mixture.

Placebo and vit D3-loaded emulsions, maintained at room temperature under continuous stirring, were pumped by air pressure (P = 330 mbar) through a nozzle 450 μm in diameter. The vibration frequency used to break up the laminar liquid jet was set at 750 Hz. The falling droplets entered the gelling bath (100 mM calcium chloride aqueous solution), where they were maintained for 15 min; then they were collected by filtration, washed with deionized water, and dried for 2 h by dynamic drying in a fluid bed dryer under airflow at 27 °C.

Being vit D3 susceptible to light degradation, many precautions were taken to preserve its stability.

#### 4.2.5. Determination of Residual Water Content of Dried Microparticles

The percentage of residual water in dried microparticulate systems was indicative of the efficiency of the drying process. In particular, the amount of residual water present in dried microparticles was determined by a thermobalance (Radwag—Ma50/1.R.WH). A small sample was placed on the weighing pan of the balance, and the final temperature, set at 125 °C, was reached gradually and maintained until the sample weight was constant. During the test, the balance registers the variation of sample weight attributable to water evaporation. The obtained results are expressed as the percentage of mass loss by the sample during the test compared to its initial weight.

#### 4.2.6. Morphological and Particle Size Analysis

Size and morphology of microparticles were investigated using optical microscopy (Stereomicroscope Leica-S9i). In particular, each formulation was photographed immediately after the preparation (in the swollen state) and after the drying process. The average diameter and shape factor of at least 50 units for each batch were determined using the image analysis software Image J (National Institute of Health, Bethesda, MD, USA) [26]. The shape factor is a value that provides information about the roundness of particles, the closer it is to 1, the more regular the particle is (the shape factor of a sphere is 1) [27].

Particle size distribution was defined by the sieve method: about 80 g of microparticles were put to the top of a series of sieves (1 mm, 710 and 500 µm) arranged in decreasing size apertures from top to bottom. The stack of sieves was vibrated, the amount of sample retained on each sieve was weighed and the particle size distribution curve was constructed.

Moreover, morphological evaluation of the sample surface and of the internal cross-section was carried out by scanning electron microscopy (Phenom XL, Thermo-Fischer Scientific, Waltham, MA, USA). Before analysis, which was carried out at 15 kV voltage, samples were sputter-coated with gold.

#### 4.2.7. Swelling Test

The ability of placebo and vit D3-loaded dried microparticles to absorb fluid and to swell when put in contact with water was investigated. Amounts of the different samples were weighed and introduced in a vial in which 5 mL of water was added. The vial was maintained at room temperature and after predefined time intervals (5, 15, 30, 60 and 120 min), the sample was recovered and weighed again. The equation reported below (Equation (1)) was used to calculate the swelling percentage:

Sw % = 100(Wt − W_0_)/W_0_ (1)

Wt is the sample’s weight after contact with the fluid and W_0_ is its initial weight [28].

#### 4.2.8. Flowability Test

Flowability properties of dried microparticles were assessed by the determination of the static angle of repose, according to the European Pharmacopoeia requirements [29]. The angle of repose depends on the density, surface area and coefficient of friction of the material. Powders with an angle of repose greater than 50 degrees are not adequate for manufacturing purposes. About 80 g of microparticles were introduced into the flowability tester, equipped with a 15 mm in diameter orifice and placed at 19.5 cm in height. The sample flows freely through the nozzle, forming a conical pile on the horizontal plane. The angle of repose is the angle between the oblique side of the powder cone and its base. The smaller the angle is, the better flowability properties characterize the powder [30].

#### 4.2.9. Content of Vegetable Oil Blend

The quantification of the oil content in dried microparticles (with or without vit D3) was carried out after liquid–liquid extraction with n-hexane and acetone. Briefly, 1 g of placebo or vit D3-loaded dried microparticles was dissolved in 50 mL phosphate buffer solution (0.2 M) at pH 6.8. The obtained solution was transferred into a separatory funnel and a solvent mixture composed of acetone (50 mL) and n-hexane (70 mL) was added. A vigorous mixing step was followed by a phase separation step, after which three different phases were visible in the funnel: acetonic phase, aqueous phase and n-hexane phase respectively from the bottom to the top. The hexane phase containing the vegetable oil blend was removed from the top of the funnel and restored with fresh n-hexane (50 mL) and the entire process was repeated four times.

The n-hexane phase recovered was introduced into a rotating evaporator (Buchi, Rotavapor R-210 equipped with heating bath B-491 and vacuum pump V-700) to eliminate the organic solvent. The residual oil was gravimetrically quantified.

The oil content was calculated according to the following Equation (2):

Oil % = 100(Oex/W)(2)

where Oex indicates the amount recovered by the extraction and W is the weight of the dried microparticles used.

#### 4.2.10. Determination of Vegetable Oil Blend on the Microparticle Surface

Microencapsulated systems (5 g) were mixed with 30 mL hexane using a vortex mixer for 1 min and then the solvent was recovered by filtration. The procedure was carried out twice on each sample. Hexane was completely evaporated in a rotating evaporator and the surface oil was determined gravimetrically.

#### 4.2.11. Vitamin D3 Recovery

The extraction of vit D3 from the lipid matrix was carried out by solid-phase extraction [31]. A total of 10 mg of each oily solution extracted from microparticles was diluted in 3 mL of hexane. The sample was loaded onto a Resprep NH2 SPE tube (Restek Corporation, Bellefonte, PA, USA) previously conditioned with the same solvent. After washing with 9 mL hexane, the target compound was eluted with 6 mL of ethyl acetate. Finally, the solvent was removed under nitrogen flux and the residue was dissolved in 1 mL methanol and analyzed by HPLC-DAD.

The vit D3 quantification was performed using a Shimadzu LC-20A Prominence chromatographic system equipped with a diode array detector (DAD detector SPD-M20A). Separation was performed on a reversed-phase Kinetex C-18 100 Å LC Column (50 × 2.1 mm i.d., with a particle size of 5 μm) (Phenomenex, Torrance, CA, USA), protected by a guard column containing the same phase, at 35 °C. Eluent A was water/formic acid 0.1% *v*/*v* and Eluent B acetonitrile/formic acid 0.1% *v*/*v* (HPLC grade solvents). The elution program used (total run time: 11 min, flow rate: 500 μL/min) was as follows: isocratic 60% B (1 min), from 60% to 95% B (1 min), isocratic 95% (3 min), from 95% to 60% B (1 min) isocratic 60% B for equilibration of the column (5 min). The volume injection was 5 μL. DAD detection was performed at 265 nm. The validation of the HPLC-DAD chromatographic method for the vit D3 recovery analysis has been briefly described in Table 8.

## Figures and Tables

**Table 1:** Fatty acids content of the vegetable oil blend and ω6:ω3 ratio.

Fatty Acid	Oil Blend (%) ± sd	Dried Microparticles (%) ± sd
C16:0	10.0 ± 0.18	10.30 ± 0.12
C16:1	0.32 ± 0.01	0.33 ± 0.01
C18:0	2.79 ± 0.08	2.80 ± 0.02
C18:1cis	69.90 ± 0.15	69.90 ± 0.07
C18:2n6cis	12.3 ± 0.05	12.10 ± 0.06
C18:3n6	0.49 ± 0.01	0.47 ± 0.01
C18:3n3	2.88 ± 0.01	2.77 ± 0.01
C20:0	0.56 ± 0.01	0.55 ± 0.01
C20:1	0.44 ± 0.01	0.42 ± 0.01
C22:0	0.17 ± 0.01	0.17 ± 0.01
C24:0	0.07 ± 0.01	0.08 ± 0.01
Fatty acid	Oil blend (%) ± sd	Dried microparticles (%) ± sd
ω6	12.80 ± 0.05	12.60 ± 0.05
ω3	2.88 ± 0.01	2.77 ± 0.01
ω6:ω3	4.45 ± 0.02	4.55 ± 0.02

**Table 2:** Peroxide values ± sd of the vegetable oil blend as it is and from microparticles.

	Iodometric Method	Spectrophotometric Method
Fresh vegetable oil blend	4.5 ± 0.1	8.4 ± 0.1
Vegetable oil blend extracted from microparticles	---	43.0 ± 2.1
Vegetable oil blend after solvent treatment	---	36.5 ± 3.0

**Table 3:** Characteristics of the emulsions and of the dried microparticles produced in the preliminary study.

	Emulsion Viscosity (cP)	Placebo Dried Microparticles Mean Diameter ± sd	Placebo Dried Microparticles Shape Factor ± sd
OL-1	144	1212.05 ± 226.43	0.787 ± 0.145
OL-2	956	1643.50 ± 87.28	0.923 ± 0.052
OL-3	2298	1679.47 ± 75.38	0.901 ± 0.056
OL-4	253	1515.34 ± 116.55	0.855 ± 0.101
OL-5	1426	1448.49 ± 98.35	0.916 ± 0.053

**Table 4:** Average diameters and shape factors of wet and dried microparticles.

Product	Mean Diameter Wet (µm) ± sd	Mean Diameter Dried (µm) ± sd	Shape Factor Wet ± sd	Shape Factor Dried ± sd
Placebo microparticles	1776 ± 460	1121 ± 240	0.932 ± 0.050	0.940 ± 0.036
vit D3 microparticles	1977 ± 240	1092 ± 163	0.938 ± 0.035	0.932 ± 0.052

**Table 5:** Percentages of residual water in dried microparticles and flowability properties.

Product	Residual Water (%)	Angle of Repose (°) ± sd
Placebo microparticles	6.42	27.40 ± 0.21
vit D3 microparticles	6.08	28.70 ± 1.47

**Table 6:** Percentage composition of placebo emulsions prepared in the preliminary study.

Components	OL-1	OL-2	OL-3	OL-4	OL-5
Sodium alginate Protanal LF10/60	1.419	2.578	3.329	---	---
Sodium Alginate Manucol	---	---	---	1.419	---
Sodium alginate Farmalabor	---	---	---	---	1.419
Water	94.620	90.224	87.351	94.620	94.620
Flaxseed flour	0.236	0.429	0.555	0.236	0.236
Vegetable oil blend	3.725	6.769	8.765	3.725	3.725

**Table 7:** Percentage composition of placebo and vit D3-loaded emulsions.

Components	OL-2 Placebo	OL-2 vit D3
Sodium alginate Protanal LF10/60	2.578	2.578
Water	90.224	90.224
Flaxseed flour	0.429	0.429
Vegetable oil blend	6.769	6.7688
Vitamin D3	---	0.0002

**Table 8:** Validation of the HPLC-DAD chromatographic method for the vit D3 recovery analysis.

Range (ng)	R^2^	Calibration Equation	Barlett Test (*p* < 0.05)	Shapiro–Wilk Test (*p* < 0.05)	Mandel Test (*p* < 0.05)	LOF (*p* < 0.05)	LOD (ng)	LOQ (ng)
0.5–10	0.9977	y = 5526.1x + 2439.7	0.162	0.609	0.137	0.162	2.71	8.22
20–75	0.9957	y = 6730.7 − 48,679	0.629	0.322	0.131	0.259	20.50	62.30

LOF lack of fit; LOD limit of detection; LOQ limit of quantification.

## Data Availability

Not applicable.

## Supplementary Materials

The following supporting information can be downloaded at: https://www.mdpi.com/article/10.3390/gels9030255/s1, Figure S1: stereomicroscope image of OL-2 emulsion.
