# Supplementary figures and images for: Microencapsulation of a Pickering Oil/Water Emulsion Loaded with Vitamin D3
Source: Gels. 2023 Mar 22;9(3):255. doi: 10.3390/gels9030255 (PMC10048092; doi:10.3390/gels9030255)

Supplementary materials

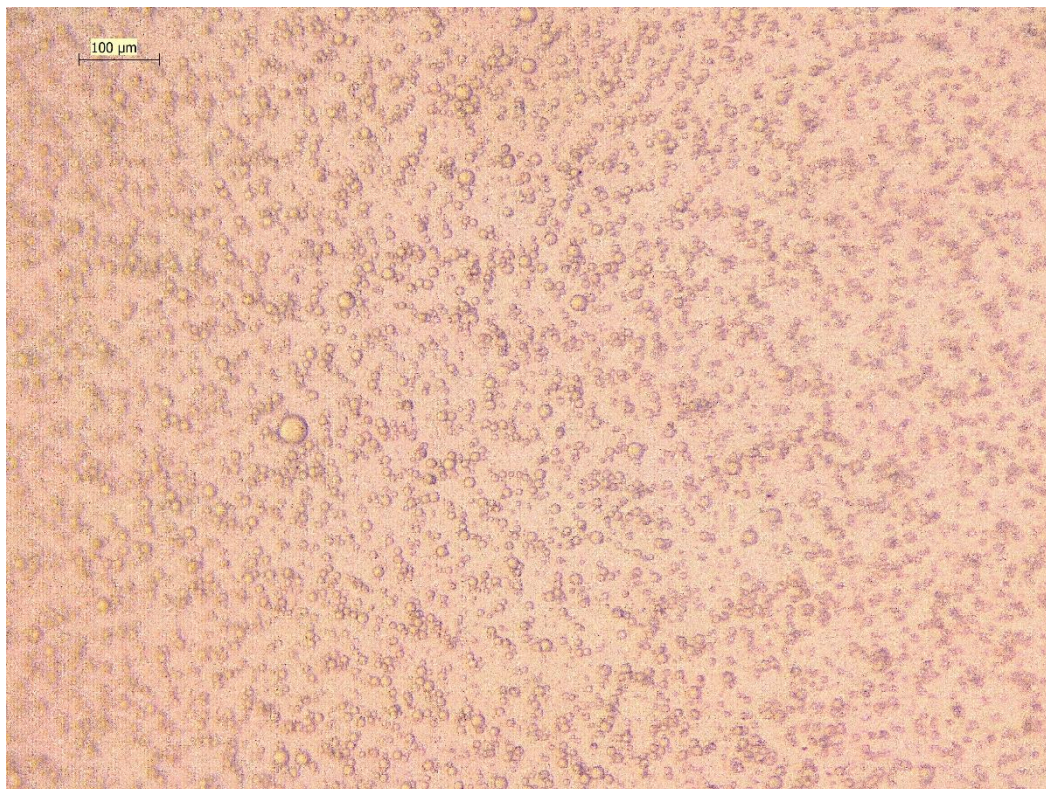

**Figure S1:** stereomicroscope image of OL-2 emulsion.

Supplement: Supplementary file 1 [file gels-09-00255-s001.zip › gels-2256024-supplementary.pdf]
